# Feeding Strategies in Newborns and Infants During the COVID-19 Pandemic—Polish Cross-Sectional Study

**DOI:** 10.3389/ijph.2023.1605590

**Published:** 2023-06-29

**Authors:** Aleksandra Wesołowska, Bartłomiej Walczak, Kinga Kalita-Kurzyńska, Aleksandra Mołas, Agnieszka Bzikowska-Jura

**Affiliations:** ^1^ Department of Medical Biology, Laboratory of Human Milk and Lactation Research, Medical University of Warsaw, Warsaw, Poland; ^2^ Human Milk Bank Foundation, Warsaw, Poland; ^3^ Institute of Applied Social Sciences, University of Warsaw, Warsaw, Poland

**Keywords:** COVID-19 pandemic, exclusive breastfeeding, infants’ feeding, infants’ nutrition, human milk

## Abstract

**Objective:** We aimed to analyze factors affecting feeding strategies of newborns and infants during the COVID-19 pandemic in Poland.

**Methods:** The cross-sectional study using a self-developed CAWI questionnaire was conducted between February and April 2021 among Polish mothers. The analysis included responses from 1,485 women who delivered during the COVID-19 pandemic restrictions. The inferential analysis uses Parson’s chi-square test and the series of logistic models. The data were weighted to adjust age and educational level distribution.

**Results:** When hospitalized, lack of skin-to-skin contact (OR = 0.094; *p* < 0.001, 95% CI [0.057 0.156]), not being informed about direct breastfeeding in the pandemic (OR = 0.195, *p* = 0.006, 95% CI [0.61 0.62]) and being suspected for COVID-19 (OR = 0.379, *p* < 0.001, 95% CI [0.223 0.642]) reduced the probability of breastfeeding. Feeding plans and feeding after leaving the hospital were impacted only by the educational level (OR = 2.463, *p* = 0.028, 95% CI [1.1 5.518]).

**Conclusion:** While the mother’s education level plays a key role in the nutrition plans and long-term feeding strategy, PUI status and hospital practices (lack of skin-to-skin and proper information) had a major negative impact on breastfeeding rates in the hospital.

## Introduction

Optimal nutrition from the beginning of life is considered as essential global health intervention to ensure long-term health and economic outcomes [1]. It is well-documented that exclusive breastfeeding (EBF) is the most appropriate way to feed a baby until the end of 6 months of age [2]. According to the definition proposed by the World Health Organization (WHO), EBF concerns a situation in which a child receives only human milk (HM) from breastfeeding mother or expressed own mother milk/donor milk or a wet nurse and no other solids or liquids except for medicines, drops or syrups consisting of minerals or vitamins for the first 6 months of life [3]. In this context, breastfeeding (BF) is defined as the provision of own mother’s HM regardless of how it is delivered to the baby (by putting baby into the breast or feeding by bottle with fresh expressed milk) [4]. Despite the WHO recommendations and the nutritional and health benefits of EBF, worldwide, early initiation of BF—within 1 hour of birth was introduced only in 39% of cases and only about 44% of newborns and infants aged 0–6 months were EBF over the period of 2015–2020 [5]. What is worse, during crises and pandemics, feeding strategy is one of the daily practices whose quality declines dramatically [6, 7]. As it was reported by Koleilat et al. [8] BF rates in low-income population significantly decreased during COVID-19 pandemic from 64.6% to 56.8% and from 48.7% to 38.6% at three and six months, respectively. Additionally, the percentage of EBF infants significantly decreased at all time points (one, three and six months). In Europe, dependent on country, directly after birth, between 56% and 98% of infants were reported to receive any HM, and at 6 months their shares ranged between 38% and 71%. Additionally, from 13% to 39% of infants were breastfed or exclusively breastfed, respectively [9]. In crisis situations, the deterioration of nutrition quality of infants and young child nutrition and exposure to malnutrition is associated not only with the breakdown of the supply chain and improper sanitation, but also with a decline in maternal confidence and emerging barriers to initiating and continuing BF.

There is a significant concern that COVID-19 responses have had a negative impact on the nutritional strategies of babies born to women exposed to SARS-CoV-2 infection. Skin-to-skin contact (SSC) and rooming-in are two practices supporting EBF that were temporarily banned during the COVID-19 pandemic, mainly due to concerns about transmission of the virus from an ill mother to her baby [10].

Therefore, the present study aimed to find the factors influencing the choice of newborns and infants nutrition made by women giving birth during the COVID-19 pandemic. We considered three sensitive periods: before the baby is born, during the neonatal period and during infancy, to analyze how making this decision affects the child. The decision made at each of these stages may trigger short- and long-term consequences because both the amount of HM and the timing of the supply have health implications.

## Methods

### Study Design and Participants

The methodology description follows the STROBE guidelines for a cross-sectional studies [11]. This survey was conducted between February and April 2021 in Poland among mothers of babies born during the COVID-19 pandemic. The study group involved women who expressed their willingness to complete the questionnaire with writing and reading skills in Polish. In the further analysis, we limited the sample to women who delivered after 10th March 2020 when the COVID-19 pandemic was recognized in Poland. There were no other exclusion criteria. The time period the respondents’ babies birth included the first and second waves of pandemic in Poland. The questionnaire was piloted in February 2021 on a sample of 21 patients with documented SARS-CoV-2 infection who delivered their babies in Polish hospitals between May 2020 and September 2020.

Considering the extraordinary conditions caused by the COVID-19 pandemic, the survey was carried out using the CAWI method (Computer-Assisted Web Interview). The final CAWI questionnaire was created on SurveyMonkey platform, and the link to the survey was shared via social media (Facebook, Instagram) by Human Milk Bank Foundation (HMBF), Polish non-governmental organization. The target group of HMBF social media includes mothers of preterm and children staying in the hospital, women at the time of pregnancy and delivery, and breastfeeding mothers.

The following study was performed in compliance with the principles outlined in the Declaration of Helsinki and approved by the Bioethical Committee of the Medical University of Warsaw in Poland. Participation of respondents was voluntary and anonymous.

### Research Tool

The tool used for the purpose of this study was a self-developed questionnaire including 38 questions concerning demographics, delivery and fertility data, COVID-19 status in the perinatal period, clinical practices supporting BF, infant nutrition, lactation support and information given by medical staff. The complete version of the study tool can be found in Supplementary Table S1.

Compliance with breastfeeding‐related policies and practices in maternity wards has been assessed with the questions based on self–appraisal tool BFHI “Section Hospital Self—Appraisal and Monitoring” World Health Organization/UNICEF 2009 [12]. The questions directed to mothers measured all the principles for the “Ten Steps to Successful Breastfeeding” implemented by WHO’s Baby-Friendly Hospital Initiative, which is considered the gold standard of breastfed childcare [13].

### The Characteristic of Feeding Strategies

**FIGURE 1:**
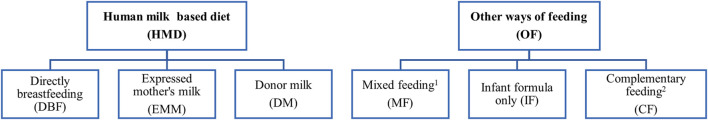
Feeding strategies of newborns and infants (Poland, 2021). ^1^ Mixed feeding - human milk based diet (HMD) + infant formula (IF). ^2^ Complementary feeding - mixture of human milk based (HMD) diet and/or infant formula (IF) and other additional food products such as: water, juice, tea; solid, soft or semi-liquid foods. Source: Survey on newborns and infants feeding in COVID-19 pandemic, Poland. 2021.

In the analysis, we aimed to identify the factors influencing the choice of newborns and infants’ feeding strategy, made by mothers who gave birth to babies during the COVID-19 pandemic. We used the self-developed typology of feeding strategies presented in Figure 1.

### Data Analysis

The analysis combines two strategies: descriptive and inferential. For the first one, frequencies for the ordinary and nominal variables and central tendencies/dispersion values for the discrete variables are provided. The inferential analysis uses Parson’s chi-square test and three series of logistic models, with outcome variables and predictors shown in Table 1. The data were weighted to adjust age and educational level distribution. Population data were obtained from the census data of the Polish Statistical Office (GUS).

**TABLE 1:** The structure of the models used in the analysis (Survey on newborns and infants feeding in COVID-19 pandemic, Poland. 2021).

Model	Outcome variable		Predictors
	Value 1	Value 0	
Nutrition plans in pregnancy (*n* = 1,381)			
	DBF^a^	IF^b^ or MF^c^	Previous baby feeding experiences
			Mother’s educational level
			Mother’s age
			Time after the delivery (days)
Nutrition in a hospital (*n* = 1,275)			
2a	DBF	IF	Skin-to-skin contact experiences after the delivery
2b	HMD^d^	IF	COVID-19 status getting information about the risks and benefits of breastfeeding during COVID-19 pandemic
2c	HMD	IF or MF	Mother’s educational level
			Mother’s age
			Time after the delivery (days)
Nutrition within the last 24 h (only babies between two and 6 months, *n* = 585)			
3a	DBF	IF or MF or CF^e^	Previous baby feeding experiences
3b	HMD	IF or MF or CF	Skin-to-skin contact experiences after the delivery
			COVID-19 status getting information about the risks and benefits of breastfeeding during COVID-19 pandemic
			Lactation support in hospital
			Information about lactation support after leaving the hospital
			Mother’s educational level
			Mother’s age

^a^ DBF, Direct breastfeeding.

^b^ IF, Infant formula.

^c^ MF, Mixed feeding.

^d^ HMD, Human milk-based diet.

^e^ CF, Complementary feeding.

The analysis was performed with SPSS ver. 28 software package. However, due to the non-random sampling, results other than descriptive should be treated with caution.

## Results

### General Characteristic of the Study Participants

In total, 1,515 women submitted a questionnaire, whereas 30 responses were excluded as delivery dates were before the 10th of March 2020, when the COVID-19 pandemic was recognized in Poland and epidemic restrictions was implemented. The age of respondents ranged from 17 to 44 years (M = 29.85 years, Mdn = 29 years, SD = 3.94). More than 60% of participants declared at least a master’s degree and almost 18% held a bachelor’s degree. The data presented in the following sections are weighted to adjust the over-representation of women with university degrees and disproportions in age.

Almost half (47.5%) of respondents were tested for COVID-19 during admission for delivery. Most person under investigation (PUI) [14] (85.9%) had negative COVID-19 test results.

Slightly more than half of the surveyed women had a vaginal delivery (56.5%) and had only one child (60.6%). The share of C-sections among women diagnosed for COVID-19 was higher (48.6%) than among the non-diagnosed ones (39.0% χ^2^_(2, n=1171.3)_ = 11.032; *p* = 0.004). Considering the mothers who have older children (n unweighted = 517), most of them (91.1%) have had some previous BF experience. Detailed general characteristic of the participating women is presented in Supplementary Table S2.

The respondents were also asked about the length and quality of SSC in an immediate postpartum period. Women untested for COVID-19 more often experienced the first contact with their infant immediately after childbirth compared to women investigated for COVID-19 (57.6% versus 50.9%). Over one-third of PUIs for COVID-19 (35.4%) did not experience any SSC with their children, while non-tested women had a lower share by 7.1 pp. (χ^2^_(2, n weighted=1171)_ = 7.127; *p* = 0.028). More mothers untested for COVID-19 (55.3%) indicated that SSC duration was 1–3 h than in the case of mothers suspected of the disease (41.8%, χ^2^_(4, n weighted=802)_ = 18.169; *p* = 0.001). Diagnosed women were more likely (by 10.9 pp) to be separated from their newborns during hospitalization (χ^2^_(2, n weighted=1171)_ = 37.249; *p* < 0.001). Detailed data concerning SSC is presented in Supplementary Table S3.

### Newborn’s Feeding Plans

The vast majority (95%) of women who answered this question (n weighted excl missing answers = 1,129) planned to feed their baby HMD while they were pregnant. 2.8% declared MF, 0.5% - IF. A small percentage (1.7%) of mothers did not think about this issue during pregnancy. Changes in plan caused by the COVID-19 pandemic were over twice more frequent among diagnosed (8.6%) than undiagnosed (3.3%) mothers (χ^2^_(1, n weighted=1108)_ = 14.324; *p* < 0.001).

The first model examines possible determinants of DBF plans. We used previous BF experiences, educational level, maternal age and as predictors. The outcome variable was the baby feeding plan (DBF vs. IF or MF). As the Hosmer-Lemeshow goodness of fit test indicates, the model was well fitted (χ^2^_H-L(8)_ = 3.808, *p* = 0.874), but its quality was low (R^2^_N_ = 0.05). There were two significant predictors related to experience with BF and maternity itself and one to the educational level. Primiparous women were 3.3 more likely to plan DBF (OR = 3.318, *p* = 0.044, 95% CI [1.035 10.636]). The experience of previous DBF was a stronger predictor than being primiparous. If a woman breastfed her older child, the probability of having DBF plans increased almost four times (OR = 3.672, *p* = 0.041, 95% CI [1.053 12.802]). Women who obtained BA or MA diplomas were over two times more likely to plan DBF (OR = 2.285, *p* = 0.032, 95% CI [1.074 4.861]). Detailed results are presented in Table 2.

**TABLE 2:** Feeding plans declared by participating mothers (Survey on newborns and infants feeding in COVID-19 pandemic, Poland. 2021).

		B	SE	OR	95% CI
Did you breastfeed your older child?	No, I did not	—	—	—	
	Yes, I did	1.301	0.637	3.672*	1.053
					12.802
	No, it is my first child	1.199	0.594	3.318*	1.035
					10.636
Educational level	University level	0.826	0.385	2.285*	1.074
					4.861
Age (years)		0.036	0.046	1.037	0.948
					1.134
Time after the delivery (days)		0.002	0.002	1.002	0.999
					1.006
Constant		0.180	1.424	1.198	

Dependent variable: baby feeding plans when pregnant (1-DBF, 0–IF or MF).

*p < 0.05 **p < 0.05, *n* = 1,381.

### Newborn’s Feeding Strategy in the Hospital

More than half of the women who answered the question (54.9%) about feeding choice in hospital setting have indicated exclusive DBF. The share of exclusive EMM was low (3.0%), DM – scarce (0.2%, two respondents only). Only 3% combined different HMD, two-thirds of whom (2.1% of valid answers) merged DBF and EMM. Among responses received, 11.7% indicated IF without any form of HM, while 27.3% of the women used MF. Comparing these results with the question about feeding plans when pregnant, we may find that 36.3% of women who planned HMD and answered the question about feeding in the hospital changed their infants feeding method.

Model 2a examined possible determinants of DBF versus using IF in hospital settings. We used SSC experience, mother status (PUI or not tested for COVID-19), being informed about risks and benefits of DBF in the pandemic, declared educational level, age and the time from the delivery. The model fitted well with the data (χ^2^_H-L(8)_ = 6.096, *p* = 0.636), and explained a 35.5% of probability of change in DBF (R^2^_N_ = 0.355). Three predictors proved significant (*p* < 0.05). Lack of SSC, even delayed, reduced the probability of DBF by 90.6% (OR = 0.094, *p* < 0.001, 95% CI [0.057 0.156]). Being informed about the risk of DBF without complementary information about benefits reduced the likelihood of DBF by 80.5% (OR = 0.195, *p* = 0.006, 95% CI [0.61 0.62]). Moreover, the women diagnosed for COVID-19 were 62.1% less likely to DBF (OR = 0.379, *p* < 0.001, 95% CI [0.223 0.642]) than those not tested for SARS-CoV-2.

For the next model (2b) we contrasted feeding the newborn with HMD with IF. The predictors remained unchanged. The model fitted well (χ^2^_H-L(8)_ = 6.365, *p* = 0.606), while its overall quality was slightly lower than the previous one (R^2^_N_ = 0.312). Three predictors, which proved significant in model 2a, were also significant here, but their influence was altered. The strongest predictor was SSC. Lack of SSC reduced the probability of feeding the infant with HMD by 88.4% (OR = 0.116 *p* < 0.001, 95% CI [0.071 0.189]). Being informed about the risks but not about the benefits of BF decreased this probability by 84.9% (OR = 0.151, *p* < 0.001, 95% CI [0.052 0.44]. Finally, PUI status reduced it by 58.5% (OR = 0.415, *p* < 0.001, 95% CI [0.249 0.691]).

The third model (2c) included feeding infants with HMD as opposed to using IF solely or MF as an output variable. Although the model fitted well (χ^2^_H-L(8)_ = 4.464, *p* = 0.813), its quality dropped when compared with models 2a and 2b. It explained only 8.7% of the changes in the probability of switching from HMD to MF (R^2^_N_ = 0.087). Two significant predictors were SSC indicators—neither PUI status nor information provided was significant in this model. No SSC reduced the probability of HMD by 68.1% (OR = 0.319, *p* < 0.001, 95% CI [0.229 0.444]) compared to immediate or delayed SSC. Delayed SSC decreased the likelihood by 54.4% (OR = 0.456 *p* < 0.001, 95% CI [0.306 0.680]). All results for models 2a-2c are presented in Table 3.

**TABLE 3:** Newborn’s feeding strategies in hospital settings (Survey on newborns and infants feeding in COVID-19 pandemic, Poland. 2021).

		Model 2a Dependent variable: newborn feeding in the hospital (1-DBF, 0–IF)				Model 2b Dependent variable: newborn feeding in the hospital (1-HM, 0–IF)				Model 2c Dependent variable: newborn feeding in the hospital (1-HM 0–IF or MF)			
		B	SE	OR	95% CI	B	SE	OR	95% CI	B	SE	OR	95% CI
What was your first contact with your child like?	SSC directly after delivery	—	—	—	—	—	—	—	—	—	—	—	—
	SSC more than 5 minutes after delivery	−0.109	0.470	0.897	0.357	−0.033	0.466	0.967	0.388	−0.785	0.204	0.456**	0.306
					2.254				2.413				0.680
	No SSC	−2.360	0.256	0.094**	0.057	−2.151	0.249	0.116**	0.071	−1.144	0.169	0.319**	0.229
					0.156				0.189				0.444
Have you been tested for COVID-19 (1—yes)		−0.971	0.270	0.379**	0.223	−0.879	0.260	0.415**	0.249	−0.101	0.163	0.904	0.657
					0.642				0.691				1.243
Have you been informed about the risks and benefits of breastfeeding during COVID-19 pandemic?	Yes, I have been informed about risks and benefits	—	—	—	—	—	—	—	—	—	—	—	—
	Yes, I have been informed about risks, but not about benefits	−1.635	0.590	0.195*	0.061	−1.893	0.547	0.151**	0.052	−0.181	0.585	0.834	0.265
					0.620				0.440				2.629
	Yes, I have been informed about benefits, but not about risks	−0.285	0.746	0.752	0.174	−0.649	0.718	0.523	0.128	0.302	0.580	1.353	0.434
					3.248				2.136				4.216
	No, I have not been informed	−0.121	0.382	0.886	0.419	−0.407	0.366	0.665	0.325	−0.233	0.244	0.792	0.492
					1.874				1.363				1.278
Educational level	University level	−0.003	0.296	0.997	0.558	−0.044	0.286	0.957	0.546	−0.337	0.202	0.714	0.480
					1.779				1.677				1.062
Age (years)		0.048	0.031	1.049	0.988	0.036	0.030	1.037	0.978	−0.008	0.019	0.992	0.956
					1.114				1.099				1.029
Time after delivery (days)		0.002	0.001	1.002	0.999	0.002	0.001	1.002	0.999	0.001	0.001	1.001	0.999
					1.004				1.004				1.002
Constant		1.630	0.979	5.105	—	2.298	0.944	9.951	—	1.869	0.617	6.482	—

*p < 0.05 **p < 0.05 SSC – skin-to-skin, *n* = 1,275.

### Feeding the Child Within the Last 24 Hours Before Submitting the Questionnaire

The majority (71.1%) of mothers of children aged between two and six months who answered this question declared DBF; 3.2% used EMM exclusively. 4.8% combined DBF and EMM. One per twenty (5%) decided to limit the diet to IF, while 8.2% combined HM and IF. A smaller share (5.6%) used HM together with CF. Other solutions were scarce. 1% used HM, IF and CF together. A similar percentage (0.9%) based their children’s diet on IF and CF. One woman declared that she feeds her baby only with CF.

We used a logistic regression model (3a) to check possible determinants of DBF beyond hospital stay. This analysis concerns infants between two and six months of age at the time of conducting the survey (between 31 and 180 days during the survey completion, n unweighted = 585 after excluding missing answers). The following predictors were used: the way the older child was fed, SSC after the delivery, PUI status, getting information about the risks and benefits of BF during the COVID-19 pandemic, level of lactation support in hospital, mother’s education level, and age. The model was well fit to the data (χ^2^_H-L(8)_ = 4.447, *p* = 0.815), but its quality was low (R^2^_N_ = 0.104). There was only one significant predictor. Having the university degree increased the probability of DBF by 2.46 times (OR = 2.463, *p* = 0.028, 95% CI [1.1 5.518]).

The next model (3b) estimates the probability of feeding the baby with HMD versus IF, MF or CF. The model was well-fitted (χ^2^_H-L(8)_ = 2.06, *p* = 0.99), and was of a slightly better quality than 3a, explaining 11,2% of the probability of changes between the values of the output variable (R^2^_N_ = 0.112). Two predictors resulted in being significant. Similarly to 3a, mothers with a university degree were 2.4 times more likely to feed their children with human milk (OR = 2.541; *p* = 0.032; 95% CI [1.082 5.555]. The second significant predictor was PUI status. Being tested for COVID-19 increased the probability of feeding a baby with HMD by 99% (OR = 1.989; *p* = 0.04; 95% CI [1.033 3.83].

Results for models 3a and 3b are presented in Table 4.

**TABLE 4:** Feeding strategies of infants (Survey on newborns and infants feeding in COVID-19 pandemic, Poland. 2021).

		Model 3a Dependent variable: baby feeding within last 24 h (1- DBF, 0–IF or MF or CF)				Model 3b Dependent variable: baby feeding within last 24 h (1 - HM, 0–IF or MF or CF)			
		B	SE	OR	95% CI	B	SE	OR	95% CI
Did you breastfeed your previous child?	No, I did not								
	Yes, I did	0.887	0.708	2.429	0.606	0.671	0.737	1.956	0.461
					9.728				8.290
	No, it is my first child	0.318	0.652	1.375	0.383	0.041	0.681	1.041	0.274
					4.935				3.953
What was your first contact with your child like?	SSC directly after delivery								
	SSC more than 5 minutes after delivery	−0.445	0.461	0.641	0.260	−0.571	0.464	0.565	0.227
					1.582				1.405
	No SSC	−0.543	0.394	0.581	0.268	−0.556	0.401	0.574	0.262
					1.257				1.258
Have you been tested for COVID-19 (1—yes)		0.565	0.328	1.759	0.924	0.688	0.334	1.989*	1.033
					3.347				3.830
Have you been informed about the risks and benefits of breastfeeding during COVID-19 pandemic?	Yes, I have been informed about risks and benefits								
	Yes, I have been informed about risks, but not about benefits	−1.093	0.972	0.335	0.050	−1.276	0.987	0.279	0.040
					2.254				1.933
	Yes, I have been informed about benefits, but not about risks	−0.102	1.090	0.903	0.107	−0.243	1.103	0.784	0.090
					7.638				6.816
	No, I have not been informed	−0.011	0.491	0.989	0.377	−0.088	0.511	0.916	0.337
					2.590				2.491
Did the hospital staff offer you help with breastfeeding from the first feeding?	Yes, in the first 6 h after giving birth								
	Yes, more than 6 h after giving birth	−0.134	0.427	0.875	0.379	−0.219	0.433	0.803	0.344
					2.021				1.875
	No, the hospital staff did not offer to help me with this	−0.167	0.381	0.847	0.401	−0.190	0.389	0.827	0.386
					1.788				1.771
Educational level	University level	0.902	0.411	2.463*	1.100	0.897	0.417	2.451*	1.082
					5.518				5.555
Age (years)		0.003	0.042	1.003	0.923	−0.003	0.043	0.997	0.917
					1.090				1.084
Constant		−0.198	1.432	0.821		0.340	1.460	1.406	

*p < 0.05 **p < 0.001 *n* = 585.

## Discussion

This study showed that the most important maternal factors influencing the plans concerning babies’ nutrition strategy were previous experiences with BF (increased the probability of DBF by almost four times) and the educational level (women with university degree were over two times likely to plan DBF). Considering hospital practices, SSC was a crucial determinant for feeding practices not only for newborns but also for infants. Lack of SSC reduced the probability of DBF by almost 91%. Additionally, we observed that it was only one significant determinant of DBF beyond hospital stay—having university degree, which increased the probability by 2.46 times.

To protect children’s right to optimal nutrition, in 2002 WHO/UNICEF developed a Global Strategy for Infant and Young Child Feeding (IYCF) [15] and provided a tool for monitoring implementation of this strategy—The World Breastfeeding Trends Initiative (WBTi) [16]. The main aim of this policy was to identify improvements and gaps, as well as action that should be carried out to enhance these practices. In 2020, Zakarija-Grković et al. [17] published the results of research which aimed to describe the state of implementation of the Global Strategy IYCF in Europe. For that time, 18 WHO/EURO Member States have conducted an assessment and provided a report. Unfortunately, Poland was not included in this analysis, because in our country, BF rates and other data concerning IYCF practices are not collected and reported by authorities entities. However, hospital practices conducive to breastfeeding, such as SSC, early initiation of BF, and lactation care are guaranteed from 2018 by the Organizational Standard of Perinatal Care, a Decree of the Minister of Health [18]. Poland lacks a system for monitoring the elements of the implementation of the Organizational Standard of Perinatal Care, and the BIHF hospitals in Poland have been limited to only 96 hospitals across the country, of which 1/3 have waived the reassessment required every 5 years (unpublished data of Committee for Breastfeeding Promotion).

Nontheless, some Polish NGOs (e.g., Childbirth with Dignity Foundation) periodically conduct online surveys, thanks to which they collect and report data on women’s experience of childbirth. One of the example is “The perinatal care during the COVID-19 pandemic in the light of the experiences of women and the medical staff” [19], which revealed that during the pandemic more than 90% of women (*n* = 10,257) had skin-to-skin contact after a vaginal birth.

One of the indicators involved in WBTi is “mean duration of breastfeeding,” which, according to Zakarija-Grković et al. was the most poorly rated IYCF practice. BF duration rates varied significantly, between 3 months (United Kingdom) and 17 months (Turkey). The average median duration of BF in the assessed countries was 8.7 months. Poland lacks data on national breastfeeding rates. The data we have are fragmentary and come mainly from regional studies. However, they indicate suboptimal nutrition of newborns and infants and a relatively high percentage of children fed by formula already in the hospital (25% of newborns) and a low percentage of infants exclusively breastfed at 6 months of age (4%) [20].

Another important WBTi indicator is “infant feeding during emergencies,” which is crucial considering the increasing frequency of natural disasters, pandemics and military conflicts. The global Operational Guidelines on Infant Feeding in Emergencies (IYCF-E) [21] were provided in 2017 by Emergency Nutrition Network, IFE Core Group. Before COVID-19 pandemic, the only European country that had a national policy on IYCF-E was North Macedonia. To date, to the best of our knowledge other four countries (Croatia, Italy, UK and Poland) have prepared or are preparing (work in progress) appropriate strategy, focusing on the context of emergency situations.

Undoubtedly, the COVID-19 pandemic has challenged protecting, promoting and supporting BF, in the face of managing the risk of a fatal infectious disease. Early in the pandemic, the WHO evaluated that the risks of not BF outweigh the risks of SARS-Cov-2 infection, and therefore, recommended that regardless of COVID-19 infection status, all mothers should be encouraged to BF [22–24]. These recommendations include all practices supporting BF, like SSC, DBF within the first hour after delivery, and rooming-in. Despite these unambiguous recommendations, in many countries completely divergent clinical practices were widespread [25]. Vu Hoang et al. [7] analyzed guidance documents from 33 countries on the care of infants whose mothers were confirmed or suspected of COVID-19. They found recommendations against practices supportive for BF were common, even in countries with high infant mortality rates. What is more, in any country all aspects of WHO guidance were recommended. DBF was recommended in 48% (confirmed) and 45% (suspected) of countries. In turn, early initiation of BF was allowed only in seven countries (21%). In Poland, the standard of care in case of delivery of a mother with COVID-19 or risk of infection has been changed four times during the first few months of the pandemic. The initial recommendations (March/April 2020) were very restrictive. Considering mothers confirmed or suspected of COVID-19 DBF and feeding with expressed milk, were discouraged and the isolation of the mother from the baby was recommended [26]. It was only 5 months (September 2020) of the pandemic when the recommendations changed, and mothers were allowed to breastfeed. None of the recommendations and guidelines applicable at that time referred directly addressed the management of mothers uninfected with SARS-CoV-2. Thereofre, it should be considered that there were no restrictions in this regard and the Organizational Standard of Perinatal Care was fully applicable to healthy mothers giving birth during the pandemic.

Many studies from different countries [27–29] revealed that the COVID-19 pandemic had led to changes in BF rates (initiation and duration of BF). Even if pregnant women planned to breastfeed their babies, stress, uncertainty of the pandemic and various ambiguous recommendations concerning feeding practices could lead to altering their previous decisions. In a study from New York City [29], 35.3% of mothers indicated that the pandemic, mainly the separation and subsequent difficulties with getting the baby latch to the breast, were the main reasons for the change in feeding strategy from pre-delivery to hospital or home decisions. Regarding pre-delivery plans, 60% of respondents intended to breastfeed, whereas in fact, only 9% and 19% of babies were breastfed at hospital and at home, respectively. In turn, in our study, we observed that during pregnancy 95% of mothers planned to use own milk (BF or EMM) and 8.6% of diagnosed women declared that the pandemic caused changes in their feeding strategy. Unfortunately, only 65% of mothers who planned to use HMD despite the COVID-19 pandemic managed to avoid their newborn formula supplementation in hospital. The formula introduction correlated with maternal PUI status. Considering that a national recommendation allowing breastfeeding by women suspected of COVID-19 was issued in Poland in September 2020, we can assume that supplementation was not due to maternal choice but rather to COVID-19-related restrictions. According to the Organizational Standard of Perinatal Care, the administration of the formula can take place in the hospital for medical reasons, but the mother must give her consent. During the COVID-19 pandemic, some hospitals reversed this rule and COVID-19-positive mothers had to sign a consent form for the administration of their own milk [30]. What is more, we found that if a woman breastfed her older baby, the probability of having DBF plans increased almost four times. It indicates that the strongest predictor was probably connected with a positive experience with BF.

Considering that BF is vicinity- and touch-dependent behavior, even minor changes in practices concerning rooming-in (e.g., maintenance of a minimum 2 m distance from the newborn) could compromise the establishment of BF [23]. Ball et al. [31] performed a randomized trial of infant sleep location in the postnatal ward and observed that newborns sleeping in close proximity to their mothers (bedding‐in) facilitate frequent feeding in comparison with rooming‐in practices (stand-alone cot condition). In our study, 35.4% women diagnosed for COVID-19 had no SSC with their babies and other 13.7% of cases did not have the opportunity of SSC immediately after delivery, but minimum several minutes later. Additionally, we reported that lack or delayed SSC reduced the probability of BF in hospital settings by almost 91%. Similar results were obtained for predictors related to feeding the infant with HMD in general. The strongest predictor in this analysis was SSC. These observations are consistent with results from the American study mentioned before [29]. In the study sample of 85 mothers, 58% were separated from their newborns immediately after birth. Worse, none of them was able to initiate BF in hospital and only 12% breastfed their babies after coming home. Contrary, in the same study, 22% of non-separated mothers initiated BF in the hospital settings and 28% breastfed when they arrived home. In the present study, most women who answered this question (54.9%) declared DBF in the hospital and 27.3% used the mixed method (HMD + IF). We also reported that being informed about the risks but not about the benefits of BF when suspected of COVID-19 decreased the probability of feeding a baby with HMD by 80.5%. This observation is consistent with the Breastfeeding Report Card (the United States 2020) [32], indicating that hospital practices are crucial for initiating and establishing BF. It is also underlined that individualized support in the first few hours and days is critical to help mothers meet their BF goals.

Our last analysis involving infants between two and 6 months of age (n = 549) revealed that most were directly breastfed beyond hospital settings and that maternal university degree increased the probability of DBF by almost 2.5 times. In 2021 Neves et al. [33] performed an analysis concerning maternal education and equity in breastfeeding and involved data obtained between 2000 and 2019 from 81 countries. The authors observed increases in prevalence for early initiation of BF (in women with no formal education) and exclusive BF (in higher educated women). Interestingly, with a few exceptions, the use of IF was higher among children of women at the highest education level in all regions. Then, it is supposed the evident choice of either BF or IF among educated mothers reflects an inverse equity hypothesis [34], whereby early adopters include families with greater access to information about the benefits of BF practices and to health services that provide BF promotion, while at the same time continuing the practice of feeding with IF. A surprising result is the correlation between being tested for COVID-19 on the time of delivery and prolonged direct breastfeeding after hospital discharge. At the same time our study did not confirm the link with lactation care and information about breastfeeding benefits on increased chance to breastfeed in infancy. However, we suspect that being a PUI and the risk of being banned from breastfeeding may have motivated mothers to breastfeed. According to available data before the pandemic, many Polish mothers gave up breastfeeding not long after returning home. On the day of discharge from the hospital, 75% were exclusively breastfed, in the second month 43%, and in the fourth and sixth months 38% and 4%, respectively [20].

### Conclusion

Our results show that maternal PUI status and hospital practices, mainly lack of SSC and limited informational support, had a major negative impact on BF rates in hospital. These factors can be mediated by mothers’ previous BF experience and their educational status. Changes resulting from COVID-19 restriction of close contact negatively affected COVID-19 mothers and their newborns in hospital. However, this did not have long-lasting effects on BF maintenance and HM supply after discharge. Perhaps belief in the value of BF was crucial here, as well as suggested by the results showing that consistently unchanged decisions in pregnancy about feeding strategy are influenced by mothers’ previous experience with BF. In this context, the most important thing, aside from hospital practices, seems to be to reassuring mothers of their BF ability and making the best choice for the baby. Even more so, reliable information from staff about the benefits and risks of BF, which were not always provided in the pandemic time, can affect mother’s choice of feeding strategy.

Some limitations of this study should be noted. Firstly, in Poland data concerning newborns and infants feeding practices is not routinely collected, therefore we had no possibility to compare these results with the pre-pandemic situation. Secondly, we did not plan follow-up of this study, so the changes in feeding strategies of infants/toddlers were impossible to report. Thirdly, not all women have answered all questions which reduced the sample size for descriptive and inferential statistics and, as in every survey, this research may be affected by uncontrolled respondent bias. Finally, our population involved mothers living in Poland, mainly with university education which may decrease the representativity of the study, and caution should be used when extrapolating the results.
