# Supplementary material for: Feeding Strategies in Newborns and Infants During the COVID-19 Pandemic—Polish Cross-Sectional Study
Source: Int J Public Health. 2023 Jun 29;68:1605590. doi: 10.3389/ijph.2023.1605590 (PMC10338688; doi:10.3389/ijph.2023.1605590)
Supplement: Supplementary file 1 [file DataSheet1.docx]

**Supplementary Table 1**. Questionnaire.

| 1.What was the date of birth of your last baby? | Format: M/d/yyyy | Required  DATE BOX |
| --- | --- | --- |
| 2.When were you admitted to the hospital for the delivery of your baby? | Format: M/d/yyyy | Required  DATE BOX |
| 3.How much did your baby weigh at birth? | …………………g | Required  TEXT BOX |
| 4.How was your baby 1-2 days after birth? | 1. Healthy 2. Sick, not needing intensive care 3. Needing intensive care | Required |
| 5.What type of delivery of your baby did you have? | 1. Vaginal 2. Cesarean section with general anesthesia 3. Cesarean section without general anesthesia 4. Other | Required |
| 6.How was your first contact with the newborn? | 1. Skin-to-skin immediately after birth 2. Skin-to-skin but took more than 5 minutes after birth 3. Wrapped without much skin contact | Required  2.- branched to 7 |
| 7. If it took more than five minutes after birth for you to hold your baby, what was the reason? | 1. Because I had or was suspected of having COVID-19 2. My baby needed help/observation 3. I had been given anesthesia and wasn’t yet awake 4. I didn’t want to hold my baby or didn’t have the energy 5. I wasn’t given my baby this soon but I do not know why 6. Other (……………………………) | Linked to response 6 |
| 8. For about how long did you hold your baby this first time? | 1. Less than 30 minutes 2. 30 minutes to less than an hour 3. 1-3 hours 4. 4 hours or more 5. Can’t remember | Required |
| 9. While you were in the hospital, did your baby stay in your room day and night, except for doctor visits, bathing, or other treatments?  *Subtitle:*  *Please check the response that better describe your experience* | 1. Yes, all the time (day and night) 2. Yes, some nights but not all 3. No, my baby was not with me | Required  1, 2- skip to 11  3.- branched to 10 |
| 10. If you answer that your baby DID NOT STAY in your room?  *Subtitle:*  *Please check the response that better describe your experience* | 1. my baby was in SEPARATED room from me all the time 2. my baby was in SEPARATED room from me but not all the time 3. my baby was transferred to ANTOHER hospital 4. Other | Required  Linked to response 9 |
| ABOUT YOU | | |
| 11. How old were you when your last baby was born?  *Subtitle*  *Please write your age in numbers, e.g. 27* |  | Required  TEXT BOX |
| 12. What is your highest level of schooling? | 1. Primary 2. Junior high school/gymnasium 3. High school 4. University into bachelor degree 5. University into master degree or more | Required |
| 13. What is your job position? | 1. High-level management: CEO, president, director 2. Intellectual: teacher, scholar, artist, architect, doctor 3. Medical personnel e.g.: doctor, nurse. midwife 4. Mid-level employees: manager, specialist 5. Office staff: clerk, secretary, assistant 6. Trade and services: seller, cashier, hairdresser, waiter, sales representative 7. Company owner hiring employees 8. Company owner not hiring employees 9. Qualified personnel, e.g. in the factory, on the construction site, foreman, 10. Manual worker, e.g. cleaning work, warder 11. Farmer, agricultural worker 12. Retired, pensioner 13. Homemaker/housewife 14. Unemployed | Required |
| 1. How many children do you have (excluding this one) | I have :   1. None 2. One 3. Two 4. Three or more | Required |
| 1. Have you ever breastfed before? | 1. I breastfeed my other baby(s) 2. I did not breastfeed my older baby/babies 3. Not applicable (this is my first baby) | Required |
| 1. How did you feel when you were admitted to the hospital for the birth of your baby? | 1. Very sick  2. A little sick  3. Well, healthy | Required |
| 17. How many days did you stay in the hospital for the birth of your baby?  *Subtitle*  *From admission to discharge, e.g. 2 days... if you had to stay hospitalized due to sickness for COVID-19 include the days in the counts e.g. 7 days, 15 days...* |  | REQUIRED  NUMBER BOX |
| ABOUT THE HOSPITAL WHERE YOU HAD YOUR BABY | | |
| 18. Please select the option that best describes the hospital where you gave birth: | 1. Hospital for COVID-19 patients 2. Hospital not designated for COVID-19 3. I delivered at home 4. I don’t know | Required  3 , 4 Skip the next question |
| 19. Please write the name of the city where the hospital where you gave birth is located. | 1. TEXT BOX WITH OPTION TO SKIP 2. PREFER NOT TO SAY | TEXT BOX |
| 20. Were you tested for COVID-19 at the hospital? | 1. Yes, before admission to the hospital 2. Yes, at admission to the hospital 3. Yes, during labor 4. I was not tested 5. I don't know/I don't remember | Required  1,2,3, branched to 21  4, 5 branched to 22 |
| 21. My test for COVID-19 at the hospital was? | 1. Positive 2. Negative 3. I don't know/I don't remember | Required  1, - branched to 22,23  2, 3 –skip to 24 |
| 22.Was your baby tested for COVID-19 before discharge from the hospital? | 1. Yes 2. No 3. I don't know/I don't remember | Required  2, 3 Skip to 24 |
| 23. My baby's test for COVID-19 was? | 1. Positive 2. Negative 3. I don't know/I don't remember | Linked to response 22 |
| BREASTFEEDING |  |  |
| 24. Prior to the birth of your child, did you intend to provide breastmilk or an infant formula  product in the first six months? | 1. Breast milk (breastfeeding and/or expressed my own milk with a pump) 2. An infant formula product 3. Combination of breast milk and infant formula 4. Did not think about it prior to birth | Required  1, 2, 3 -branched to 25, 26  4.- Skip to 27 |
| 25. Did your plan for feeding your baby change due to COVID-19? | 1. Yes 2. No | Required  If Yes Click to go TEXT BOX for open-ended question  If NO Skip to 28 |
| 26. Describe how COVID-19 changed your decision about a plan for feeding your baby |  | Required  TEXT BOX |
| 27. Have you been informed at the hospital about the risk and benefits of breastfeeding your baby because you had or were suspected of having COVID-19? | 1. Yes, I was informed about risks and benefits of breastfeeding in my clinical situation related to COVID-19  2. Yes, I was informed about risks, not benefits, regarding breastfeeding in my clinical situation related to COVID-19  3. Yes, I was informed about the benefits, not risks regarding breastfeeding in my clinical situation related to COVID-19  4. No, nobody informed me about risk and benefits of breastfeeding in my clinical situation. | Required  1,2,3 - branched to 28  4 - skip to 29 |
| 28. At the hospital, who informed you about risks and benefits of breastfeeding related to  COVID-19?  *Subtitle*  *Please check all the responses that apply* | 1. Your doctor 2. Baby’s doctor 3. Nurse 4. Midwife 5. Other staff of the hospital (no mentioned above) | Linked to response 27 |
| 29. Did you sign an informed consent about breastfeeding your baby at the hospital despite risk of COVID-19 transmission? | 1. Yes, I signed my agreement to breastfeed despite risk of COVID-19 transmission  2 Yes, I signed my agreement not to breastfeed my baby because of COVID-19  3. No, nobody ask me to sign Informed consent about breastfeeding | Required |
| 30. How were you feeding your baby at the hospital? | 1. Breastfeeding, 2. With expressed milk 3. With breast milk from a human milk bank 4. With both breast milk (breastfeeding, expressed milk or milk from human milk bank) and infant formula 5. With infant formula (not breastfeeding at all) 6. Other | Required |
| 31. Did you use ANY safety measures to breastfeed your baby at the hospital?  For example, mask, wash your hands with soap or sanitizer, cleaned surfaces...) | 1. Yes 2. no | Required |
| 32. Did the staff offer you any help with breastfeeding since the first time you breastfed your baby? | 1. Yes, within 6 hours of when my baby was born 2. Yes, more than 6 hours after the birth of your baby 3. No, the staff did not offer help | Required |
| 33. Have you been given any leaflets or supplies promoting infant formula at the hospital? | 1. Leaflet with information saying breastmilk or breastfeeding may NOT BE SAFE during COVID-19 2. Leaflet with information about the types of infant formula 3. Leaflet with information saying that infant formula supports the baby’s immunity 4. Leaflet about breastfeeding with formula company logo 5. Leaflet from formula company promoting formula feeding or related supplies 6. A gift or samples to take home, including formula, bottles, or other related supplies 7. No 8. Other | Required |
| 34. Have you been given any suggestions by the staff about how or where to get help, if you have problems with feeding your baby after you return home? | 1. Yes 2. No | Required  1- branched to 35  2- skip to 36 |
| 35. if yes, where? | 1. Specialized Lactation  Consultant 2. Midwife Primary Health Care 3. Primary Care pediatrics/neonatologist 4. Other: ______ | Linked to response 34 |
| 36. Any other liquids were being given to the baby at the hospital, such as teas or sugared water? | 1. Yes 2. No | Required |
| 37. In the last 24 hours (that is, starting yesterday at the same hour I am responding the questionnaire until now), my baby received (please check all options that apply)… | 1. Breastmilk - I breastfed my baby 2. Breastmilk - my expressed milk 3. Infant formula 4. Canned or tinned milk (not infant formula) 5. Water, tea, juice, or clear broth 6. Any other liquids 7. Any solid, semi-solid or soft foods | Required |
| 38. To sum up: in your opinion, has the COVID-19 pandemic affected the situation of pregnant/postpartum women? | 1. Yes 2. No | 1- branched to 39  2- skip 39 |
| 39. Please describe how the COVID-19 pandemic has affected the situation of pregnant/postpartum women? |  | TEXT BOX |

**Supplementary Table 2**. General characteristic of the study participants.

| **Category** | **Answer** | **n (%)** | **n (%) valid** |
| --- | --- | --- | --- |
| **Age (in years)** | ≤ 19 | 4 (0.3%) | 4 (0.3%) |
|  | 20 – 29 | 716 (48.2%) | 716 (50.0%) |
|  | 30 – 39 | 694 (46.7%) | 694 (48.5%) |
|  | ≥ 40 | 18 (1.2%) | 18 (1.3%) |
|  | Missing answers | 53 (3.6%) | - |
|  | **Total** | **1485 (100%)** | **1432 (100.0%)** |
| **Level of education** | Primary education | 2 (0.1%) | 2 (0.1%) |
|  | Lower secondary education | 5 (0.3%) | 5 (0.3%) |
|  | Vocational education | 14 (0.9%) | 14 (1.0%) |
|  | Secondary education | 255 (17.2%) | 255 (17.8%) |
|  | University studies completed with a bachelor's degree | 254 (17.1%) | 254 (17.7%) |
|  | University studies completed with at the least a master's degree | 902 (60.7%) | 902 (63.0%) |
|  | Missing answers | 53 (3.6%) | - |
|  | **Total** | **1485 (100%)** | **1432 (100.0%)** |
| **Mode of delivery^1^** | Vaginal birth | 661 (56.5%) | 661 (56.5%) |
|  | Caesarean section under general anesthesia | 70 (6.0%) | 70 (6.0%) |
|  | Caesarean section with anesthesia of the operated body region | 440 (37.7%) | 440 (37.7%) |
|  | Missing answers | 0 (0.0%) | - |
|  | **Total** | **1171 (100%)** | **1171 (100%)** |
| **Number of children^1^** | 1 | 710 (60.6%) | 710 (60.6%) |
|  | 2 | 337 (28.8%) | 337 (28.8%) |
|  | 3 | 102 (8.7%) | 102 (8.7%) |
|  | ≥ 4 | 22 (1.9%) | 22 (1.9%) |
|  | Missing answers | 0 (0.0%) | - |
|  | **Total** | **1171 (100%)** | **1149 (100%)** |
| **Age of the youngest child when interviewed**  **(in months) ^1^** | up to one month (up to 30 days) | 96 (8.2%) | 96 (8.3%) |
|  | 2-6 months (31-180 days) | 549 (46.9%) | 549 (47.3%) |
|  | over 6 months (more than 180 days) | 517 (44.1%) | 517 (44.5%) |
|  | Missing answers | 10 (0.8%) | - |
|  | **Total** | **1171 (100%)** | **1161 (100%)** |
| **Previous experiences of breastfeeding^1^** | Yes, I breastfed my older child/children | 393 (33.5%) | 393 (33.6%) |
|  | No, I did not breastfed my older child/children | 38 (3.2%) | 38 (3.3%) |
|  | Not applicable (it is my first child) | 738 (63.0%) | 738 (63.0%) |
|  | Missing answers | 2 (0.2%) | - |
|  | **Total** | **1171 (100%)** | **1169 (100%)** |
| **Women diagnosed**  **for COVID-19 during the perinatal period^1^** | Yes | 556 (47.5%) | 556 (47.5%) |
|  | No | 615 (52.5%) | 615 (52.5%) |
|  | Missing answers | 0 (0.0%) | - |
|  | **Total** | **1171 (100%)** | **1171 (100%)** |
| **Women’s COVID-19  test results^1^** | Positive | 71 (6.1%) | 71 (12.9%) |
|  | Negative | 474 (40.5%) | 474 (85.9%) |
|  | I do not know / I cannot remember | 7 (0.6%) | 7 (1.3%) |
|  | Missing answers | 619 (52.9%) | - |
|  | **Total** | **1171 (100%)** | **552 (100.0%)** |

^1^ weighted data.

Source: Survey on newborns and infants feeding in COVID-19 pandemic, Poland. 2021.

**Supplementary Table 3**. The skin-to-skin contact with an infant.

| **Category** | **Answer** | **Women**  **diagnosed for COVID-19**  **n (%)** | **Women**  **non-diagnosed for COVID-19**  **n (%)** |
| --- | --- | --- | --- |
| **Skin-to-skin contact with an infant^1^*** | Skin-to-skin contact immediately after delivery | 283 (50.9%) | 354 (57.6%) |
|  | Skin-to-skin contact more than 5 minutes after delivery | 76 (13.7%) | 87 (14.1%) |
|  | No skin-to-skin contact, infant wrapped/dressed immediately after delivery | 197 (35.4%) | 174 (28.3%) |
|  | **Total** | **556 (100.0%)** | **615 (100.0%)** |
| **Duration of skin-to-skin contact with an infant^1^**** | Less than 30 minutes | 115 (32.0%) | 101 (22.8%) |
|  | More than 30 minutes, but less than an hour | 44 (12.3%) | 51 (11.5%) |
|  | 1-3 hour(s) | 150 (41.8%) | 245 (55.3%) |
|  | 4 hours and more | 44 (12.3%) | 35 (7.9%) |
|  | I do not remember | 6 (1.7%) | 11 (2.5%) |
|  | **Total** | **359 (100.0%)** | **443 (100.0%)** |
| **Day and night contact with your child during  hospitalization**  **(except for medical procedures) ^1^ **** | Yes, all the time  (day and night) | 381 (68.5%) | 497 (80.8%) |
|  | Yes, but not on all nights | 81 (14.6%) | 81 (13.2%) |
|  | No, my child was  not staying with me | 94 (16.9%) | 37 (6.0%) |
|  | **Total** | **556 (100.0%)** | **615 (100.0%)** |

^1^ weighted data.

* p<0,05 **p<0,001

Source: Survey on newborns and infants feeding in COVID-19 pandemic, Poland. 2021.
